# Endotoxin-Retentive Filters for the Online Preparation of Ultrapure Dialysis Fluid and Non-Pyrogenic Substitution Fluid: A Critical Review and Reference Guide

**DOI:** 10.3390/membranes15020051

**Published:** 2025-02-05

**Authors:** Gerardo Catapano, Giuseppe Morrone, Lilio Hu, Gionata Fragomeni, Andrea Buscaroli

**Affiliations:** 1 Department of Mechanical, Energy and Management Engineering, University of Calabria, via P. Bucci, 87030 Rende, Italy; giuseppemorrone.ic@libero.it; 2 Department of Medical and Surgical Sciences (DIMEC), Alma Mater Studiorum University of Bologna, 40138 Bologna, Italy; lilio.hu2@unibo.it (L.H.); andrea.buscaroli@unibo.it (A.B.); 3 Nephrology and Dialysis Unit, Santa Maria delle Croci Hospital, AUSL Romagna, 48121 Ravenna, Italy; 4 Department of Medical and Surgical Sciences, Magna Graecia University, Viale Europa—Loc. Germaneto, 88100 Catanzaro, Italy; fragomeni@unicz.it

**Keywords:** endotoxin retentive filter, hemodiafiltration, hemodialysis, membrane, substitution fluid, ultrapure dialysis fluid

## Abstract

Poor water treatments and concentrates to prepare dialysis fluids favor bacterial growth-producing pyrogens (e.g., endotoxins) that may cross hemodialysis, particularly high-flux, membranes. This puts hemodialysis patients at risk of acute bacteremia, pyrogenic reactions, long-term complications, loss of residual renal function, and poor nutritional status. Consequently, regulatory bodies worldwide recommend using ultrapure dialysis fluid for routine hemodialysis. Requests are also growing for the online production of sterile non-pyrogenic substitution fluid from ultrapure dialysis fluid. This way, large volumes of infusion solution may be safely and economically produced, enabling more end-stage kidney disease patients to benefit from the greater capacity of hemodiafiltration to remove toxins than purely diffusive hemodialysis treatment. Ultrapure dialysis and substitution fluids are often produced upstream from hemodialyzers by online filtration of standard dialysis fluid through cascades of bacteria- and endotoxin-retentive filters (ETRFs). Commercial ETRFs differ for membranes, operation, performance, duration and maintenance protocols, connection to a dialysis machine, disinfection procedures, and replacement schedule. Although suboptimal ETRF choice may increase treatment costs, the difficulty in gathering comparative information on commercial ETRFs complicates their selection. To aid dialysis centers in selecting the most convenient and suitable ETRF for their needs, herein, relevant characteristics of commercial ETRFs are reported and critically reviewed for a quick yet effective comparison.

## 1. Introduction

Contamination of dialysis fluids (i.e., dialysate and substitution fluid) with pathogens, such as Gram-negative bacteria, mycobacteria, fungi, molds, and endotoxins, is a serious potential threat for end-stage kidney disease (ESKD) patients. It is estimated that patients on thrice weekly maintenance hemodialysis (HD) may be exposed to volumes of dialysis fluids greater than 300 L every week, depending on the prescribed treatment dose [1,2,3]. Poor water quality, inadequate cleaning and disinfection of the water treatment and distribution systems, the concentrates used for the preparation of the dialysis fluid, and the dialysis fluid distribution systems, may favor microorganism growth and endotoxin accumulation in such fluids [3,4]. When the barrier properties of the hemodialysis membranes are not selective enough (e.g., as it could be the case of large pore membranes with a slanted intrinsic sieving coefficient spectrum) or change for external causes (e.g., dialyzer reprocessing and reuse, or inappropriate storage conditions), microorganisms and endotoxins may cross the membrane wall and enter the patient’s bloodstream, causing bacteremia, fungemia, and/or septicemia. Many pathogens whose presence in dialysis fluids or patients’ blood has been often reported (e.g., Gram-negative bacteria), and also opportunistic pathogens (e.g., some fungi), grow well in the fluids used for medical care in planktonic (i.e., suspended) form and release substances stimulating the immune system even after their death, as it is the case of endotoxins. Some form biofilms at the surface of tubing in which dialysis fluids flow, which protect them against biocidal or fungicidal chemicals. Some form spores to resist the aggression of disinfectants and sterilizers. The presence of anti-endotoxin antibodies in the plasma of hemodialysis patients [5,6] testifies that endotoxins, intact or as fragments, may cross the wall of hemodialysis membranes, more so high-flux membranes [7,8,9,10,11,12,13,14,15]. Evidence exists that endotoxin concentrations in the plasma of hemodialysis patients may increase with increasing bacterial counts in the dialysis fluid [16], causing more frequent pyrogenic reactions [17]. Coulliette et al. [2] report that six outbreaks with 177 cases were associated with endotoxin contamination, and four outbreaks with 53 cases were associated with both bacteria and endotoxin contamination, from 1973 to 1987 in the United States. Kanamori et al. report that 91% of the more recent infections were caused by Gram-negative bacteria [4]. Endotoxin contamination of dialysis fluids is believed to cause the accumulation of markers of inflammatory response (e.g., interleukin-6 and C-reactive protein) and oxidative stress, as well as of b2-microglobulin, in the serum of hemodialysis patients. This causes a chronic inflammatory state that is associated with complications typically observed in long-term hemodialysis patients, such as cardiovascular disease (the main cause of death of HD patients), loss of residual renal function, malnutrition, poor responsiveness to erythropoietin, and b2-microglobulin-associated amyloidosis, to quote a few [18,19,20].

Long-term studies show that pyrogen removal by ultrafiltration of the dialysis fluid significantly improves all such symptoms; reduces cardiovascular morbidity and mortality, the erythropoietin resistance index, and ferritin levels; and is associated with better anemia correction and preservation of residual kidney function [9,21,22,23,24]. On these grounds, regulatory bodies worldwide, such as the International Standardization Organization (ISO), the American Association for the Advancement of Medical Instrumentation (AAMI), and the Japanese Society for Dialysis Therapy (JSDT), today recommend the use of ultrapure dialysis fluid (UPDF) for routine hemodialysis [25,26,27].

Parallel to the increasing use of ultrapure dialysis fluids (UPDFs), there is an increasing request for the online production of sterile non-pyrogenic substitution fluid (NPSF) that is used as an infusate in hemodiafiltration (HDF) with high-flux dialysis membranes [28]. After some initial controversy [29], evidence has accumulated over the years showing that HDF treatments with the direct infusion into the patient’s blood of more than 23L per session of sterile NPSF improve patients’ survival; reduce the risk of all-cause and cardiovascular mortality, the number of hospitalization days, the incidence of intradialytic hypotensive events, and the decline in cognitive function; and improve dialysis-associated pathologies, such as amyloidosis and malnutrition, and patients’ quality of life [28,30,31]. Such benefits are thought to be related to the more efficient removal of middle-molecular-weight uremic toxins and markers of inflammation by HDF than HD and high-flux HD [28] and the use of dialysis fluids of higher quality. As a result, 70,000 patients were routinely treated in Europe in 2011, and more than 30,000 patients were treated in Japan in 2013. After the work of the Kidney Health Initiative on the regulatory pathways to the approval of HDF in the United States [28], the recent 510(k) clearance of the Food and Drug Administration (FDA) to machines and membrane modules for HDF suggests that soon, HDF will also be routinely used in dialysis centers in the USA [32]. A key issue to making large numbers of ESKD patients benefit from HDF is to safely produce the large volumes of substitution fluid that have to be infused in the patient’s blood and yet make the treatment economically sustainable. Fortunately, the composition of the substitution fluid does not need to be different from the dialysis fluid [28], and today, governmental regulatory bodies recommend that sterile NPSF be produced online by the ultrafiltration of ultrapure dialysis fluid [26,27].

**Figure 1:**
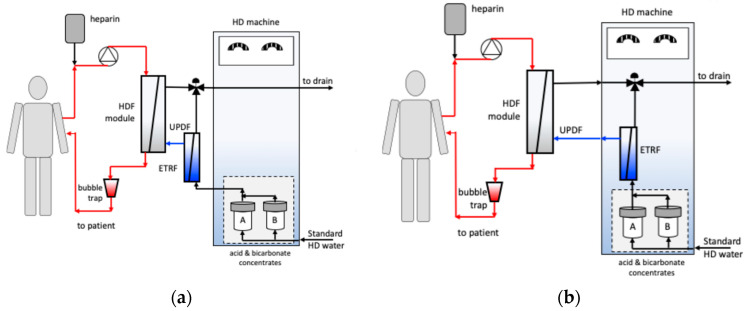

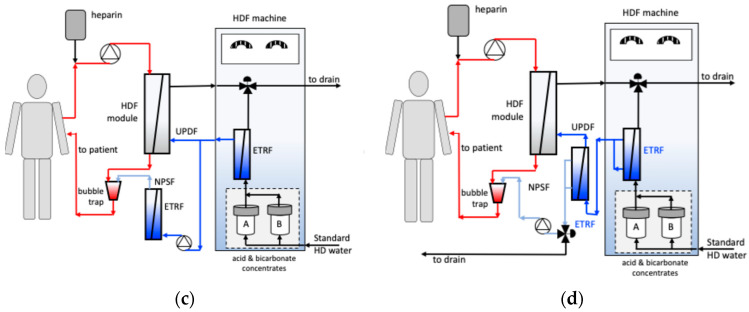
Schemes of kidney support treatments using one or two ETRFs connected differently to the dialysate pathway: (**a**,**b**) high-flux dialysis treatment using one ETRF for the production of UPDF positioned (**a**) outside or (**b**) inside the HDF machine; (**c**,**d**) post-dilution HDF treatment using two ETRFs, with the first ETRF for the production of UPDF positioned inside the HDF machine, and the second ETRF for the online production of NPSF positioned outside the HDF and connected to the dialysate pathway either (**c**) inline or (**d**) in a recirculation loop.

The current reference international standard defining the purity of dialysis fluids and indicating how to produce them from drinking water is the ISO-23500:2024, Parts 1–5 [27], an international standard that, in its previous versions, has been adopted as a whole or in part by various national regulatory bodies. Table 1 summarizes the requirements set by regulatory bodies in Europe, Japan, and the USA that the various fluids used for HD and HDF must, or are recommended to, meet. In the dialysis centers meeting the recommendations for standard dialysis water production, and where the recommended infection control and surveillance is guaranteed, ultrapure dialysis fluid and sterile non-pyogenic substitution fluid are produced in HDF machines by the online ultrafiltration of standard dialysis fluid through a cascade of at least two bacteria- and endotoxin-retentive filters (jointly referred to as endotoxin retentive filters, ETRFs) positioned in the dialysis fluid pathway. As schematically shown in Figure 1a–d, in HDF machines, ultrapure dialysis fluid is prepared online by the ultrafiltration of dialysis fluid, and that product is then split into two streams. The first UPDF stream is fed to the dialysate compartment of the HD/HDF membrane module. The second UPDF stream is ultrafiltered a second time to produce sterile non-pyrogenic substitution fluid (NPSF). The NPSF is infused in the patient’s blood during the HDF treatment in the arterial blood line (i.e., in pre-dilution mode), in the blood compartment of the HDF module (i.e., in mid-dilution mode), or in the bubble trap in the venous blood line (i.e., in post-dilution mode), the last one generally being the preferred operating mode. The first ETRF for UPDF production is positioned directly upstream from the dialysate inlet in the HD/HDF membrane module, is often incorporated in the HD/HDF machine, is disinfected with the machine, and is replaced periodically (Figure 1a,c) [28]. The second ETRF for NPSF production is positioned just upstream from the point at which the sterile non-pyrogenic substitution fluid is infused in the bloodstream, and it is often a disposable single-use ETRF that is replaced after each treatment to minimize the risks of contamination in the event of a component failure.

Although with slight differences, the current norms define an ETRF as a “membrane filter used to remove endotoxins and microorganisms from dialysis water or dialysis fluid” [26,27]. ETRFs are ultrafilters that retain bacteria, endotoxins, and their fragments primarily by size exclusion. Adsorption on the membrane pore surface contributes to enhancing the membrane endotoxin retention capacity. The bacteria and endotoxin retention capacity of ETRFs is generally expressed in terms of the logarithmic (base ten) reduction value (LRV), defined as the logarithm base ten of the challenge-to-permeate pathogen concentration ratio. LRV is the order of magnitude reduction in endotoxin or bacteria concentration that they accomplish in a standard challenge test with defined bacterial strains, media, and exposure time. It is recommended that ETRFs achieve at least an LRV = 4 for bacteria and an LRV = 2 for endotoxins to produce ultrapure dialysis fluid from standard dialysis fluid, and at least an LRV = 7 for bacteria and an LRV = 3 for endotoxins to consistently produce sterile non-pyrogenic substitution fluid from ultrapure dialysis fluid [33]. In addition to the intrinsic properties of the membranes used, the final quality of the filtered fluid depends strongly on how the ETRF is operated and on factors independent of the ETRFs, such as the quality of the drinking water, how long the filter has been used, the fluid distribution system, and the cleaning and disinfection procedure (e.g., type and concentration of the chemicals used, duration and schedule of the procedure, control, and surveillance protocols, etc.). Testing whether the microbiological purity of the produced ultrapure dialysis and substitution fluid meets the values required by the norms (Table 1) is very challenging, if not merely theoretical. It is difficult to detect quickly and accurately very small amounts of microorganisms and endotoxins with established laboratory techniques (e.g., the Limulus amebocyte lysate test in its various propositions). Moreover, ultrapure dialysis fluids and sterile non-pyrogenic substitution fluids are produced and used while the treatment is ongoing, which makes it impossible to check in laboratory tests performed ahead of their use that their quality meets the quality set by the norms [34]. For these reasons, the ISO standards acknowledge that the quality of NPSF may only be determined by its production through a validated process, provided that the system is operated and maintained according to the manufacturer’s recommendations [27]. This shifts the responsibility for ensuring the quality of dialysis fluids to the operators of the dialysis center, who have to manage all the steps of dialysis fluids preparation, as well as all the control and surveillance steps through a validated quality maintenance system [34]. The need to test the quality of the fluids produced is lifted, and the associated costs are reduced, if the EFTRs are maintained and replaced as recommended by the manufacturer. Within such a framework, using ETRFs that effectively deliver dialysis fluids meeting the requirements set by the norms, and yet fit well in the logistic and economic organization of a given dialysis center, becomes of paramount importance. This notwithstanding, gathering detailed information on the characteristics, performance, connection to HD/HF/HDF machines, disinfection procedure, and replacement schedule of commercial ETRFs is rather difficult. This difficulty complicates the thorough evaluation of the effectiveness and convenience of the ETRFs commercially available.

To bridge this gap, in this paper, a review and a critical analysis of commercial ETRFs are reported. Their most relevant characteristics are summarized in a look-up table format that may be used as a reference guide to help professionals responsible for dialysis centers quickly select the ETRFs most suited for their needs and organization. Possible directions in which ETRF operation and maintenance may be further improved to prevent the outbreak of infections, at times deadly, are then proposed as future outlooks.

## 2. Methods of Literature Search and Selection

Herein, we report a non-systematic critical analysis of the commercial and scientific literature addressing practical and theoretical aspects of the design and use of commercial endotoxin-retaining filters used in dialysis centers.

### 2.1. Inclusion and Exclusion Criteria

The analysis was restricted to the ETRFs commercially available to prepare ultrapure dialysis fluid (i.e., ultrapure dialysate) and sterile non-pyrogenic substitution fluid. Filters at the research or developmental phase were excluded from this review because their use is not yet allowed in routine dialysis practice. Information on filters for bacteria and endotoxin removal from dialysis water was not included because they more properly pertain to the equipment for water treatment systems rather than to the dialysis treatment per se. Information on intravenous endotoxin filters was not included because the design and operation of devices for treating blood are subjected to different requirements than those for treating dialysis fluids. Only ETRFs were considered for this review for which reliable information could be gathered. The features used for the analysis and reported in the tables were selected based on their subjective relevance for an informed selection.

### 2.2. Literature Search

Information on commercial ETRFs was partly obtained from publicly accessible sources (e.g., the Internet, manufacturer’s brochures or informative material, scientific papers, advertisements, articles, etc.) and partly from information provided by the manufacturers in reply to email requests. For this purpose, scientific search motors, such as MEDLINE/PubMed and Google Scholar, and general-purpose search motors, such as Google, were used. The search strategy included keywords such as “adsorption”, “bacteria”, “dialysate”, “dialysis”, “endotoxin”, “filter”, “filtration”, “fluid”, “fungi”, “hemodialysis”, “hemodiafiltration”, “infection”, “lipopolysaccharides”, “mycobacteria”, “mycotoxins”, “outbreak”, “pyrogen(s)”, “substitution”, “ultrapure”, and “waterborne”, as well as the brand name of the commercial ETRFs that were found and Boolean combinations thereof. No language restrictions were used.

Only the scientific papers that were considered relevant for the selected applications or comprehensive were accounted for in the reported analysis.

For the above reasons, the information reported below should by no means be considered exhaustive of that commercially available. Nor should the authors be held liable for the information reported or for not considering in the analysis other ETRFs than those considered.

### 2.3. Data Organization and Analysis

The data that could be gathered were organized in look-up tables to facilitate their use by the personnel of dialysis centers. In each table, the data are organized into five sections. In the Section 1, information is reported on the manufacturer, filter tradename and image, where available, and web contacts. In the Section 2, information is reported on the membrane module design and operation. In the Section 3 and Section 4, information is reported on membrane and module specifications, respectively. In the Section 5, the manufacturer’s recommendations for filter disinfection and replacement schedule are reported. In the absence of information provided by the manufacturer, the available data were critically analyzed based on that reported in scientific papers for similar membranes or membrane modules.

## 3. Specifications of Commercial ETRFs

The literature search identified about 2000 documents, including commercial brochures, technical informative material, scientific papers, and direct communications from the manufacturers. Only three out of the seven manufacturers that were contacted replied to the request for information. It was difficult to gather detailed information on the ETRFs’ features and performance, on the methods used for their characterization, and on the recommended disinfection procedures and replacement schedule from sources other than the manufacturer. For this reason, where available, we decided to report the information on the considered ETRFs directly or indirectly disclosed by the manufacturer in brochures and technical documents released in any of the countries in which a given ETRF is marketed. Reference is provided for the information gathered from scientific papers.

After checking the gathered information and eliminating duplicates, 308 documents were selected and more deeply analyzed. Information was found on 13 types of ETRFs produced by four European firms, three Japanese firms, and one US firm. Eleven types of ETRFs are marketed for the online production of ultrapure dialysis fluid. Three of them may also be used to produce sterile non-pyrogenic substitution fluid if operated in a cascade of at least two ETRFs. Most ETRFs can be cleaned, disinfected, sterilized, and reused. Two smaller-than-average ETRFs are marketed to produce online-only sterile non-pyrogenic substitution fluid in cascade with one ETRF of the larger type. They are single-use and disposable. All EFTRs marketed for the production of NPSF are designed to be placed in the dialysate pathway between the UPDF loop and the patient’s bloodline. The disposable ETRFs may be connected to the UPDF production line in a dead-end mode in a simple one inlet–one line or via a loop in which the UPDF is continuously recirculated on the upstream side of the ETRF, depending on the dialysis machine and manufacturer’s recommendations.

The requirements that the ETRFs should meet depend on their use (i.e., whether they should produce online ultrapure dialysis fluid or sterile non-pyrogenic substitution fluid) and the patient-specific clinical indications. According to that reported in [28], it was assumed that ETRFs should produce online ultrapure dialysis fluid from standard dialysis fluid at flow rates in the 600–1000 mL/min range, inclusive of that needed for the production of sterile non-pyrogenic substitution fluid. It was also assumed that ETRFs should produce non-pyrogenic substitution fluid from ultrapure dialysis fluid at flow rates in the 90–200 mL/min range. The main features and the relevant performance of the considered ETRFs are reported in Table 2, Table 3, Table 4, Table 5 and Table 6 in look-up format to permit technicians and clinicians in charge of dialysis centers to consult them quickly. In the following, the gathered information is presented and discussed in more detail.

### 3.1. Membrane Properties

All selected ETRFs are equipped with hollow fiber asymmetric membranes made of polysulfone (PSu). PSu is a hydrophobic polymer containing aromatic, sulfonyl, ether groups, and some alkyl groups. Hence, it may also be referred to as polyethersulfone (PES) or polyarylethersulfone (PAES). PSu is blended with varying amounts of water-soluble polyvinylpyrrolidone (PVP) (as in the case of Fresenius Polysulfone^®^, Medisulfone^®^, and Toraysulfone^®^ membranes), polyamide (as in the case of the Polyamix^®^ membranes by Baxter), or polyarylate (as in the case of the PEPA^®^ membranes by Nikkiso) to increase membrane hydrophilicity and permeability to water and to modulate membrane rejection and mechanical properties. In such asymmetric membranes, a highly porous sponge layer provides mechanical support to one or more selective skin layers a few tenths of a micron thick. The properties of the skin layer determine the membrane selectivity, act as a molecular sieve, and control the membrane’s hydraulic permeability.

A single skin layer generally faces the membrane lumen. The PEPA^®^ membranes feature a three-layer structure with two skin layers, one each on the membrane’s inner and outer surfaces. In most cases, the sponge layer is characterized by small spherical voids (i.e., the macropores). The Medisulfone^®^ membranes exhibit a sponge layer characterized by long finger-like voids. In either case, the size of the voids increases outwards along the membrane radius. Membrane diameter and wall thickness are generally similar to those of commercial hemodiafiltration or high-flux membranes, in particular, those produced by the same manufacturer. The CF-609N ETRF by Nipro is equipped with membranes featuring an inner diameter more than three times larger and a wall about 2.5 times thicker than the high-flux membranes produced by the same firm.

Despite this geometric similitude, the membranes used for the ETRFs feature a hydraulic permeability (i.e., the ultrafiltration rate, k_UF_, per unit membrane area) from 3 to 16 times higher than HDF or high-flux HD membranes and up to 200 times higher than low flux HD membranes. Little information is disclosed on their nominal molecular weight cut-off (NMWCO) and even less on their sieving coefficient spectrum. The available data suggest that commercial ETRFs feature an NMWCO ranging from 6000 to 30,000 [35]. No information is generally disclosed on the absolute capacity of ETRF membranes to adsorb endotoxins or pyrogens unless anecdotally and only as compared to that of high-flux HD membranes.

### 3.2. Module Design and Operation

The geometry of the ETRF closely resembles that of membrane modules for HDF, HF, and HD. A bundle of hollow fiber membranes is arranged in a cylindrical plastic housing (generally of polycarbonate or polypropylene) in a shell-and-tube configuration and is potted with polyurethane glue at its ends. Then, the membrane excess is cut away with a sharp blade, headers are glued at the module ends, and the module is equipped with connectors at the membrane lumen and shell side.

**Figure 2:**
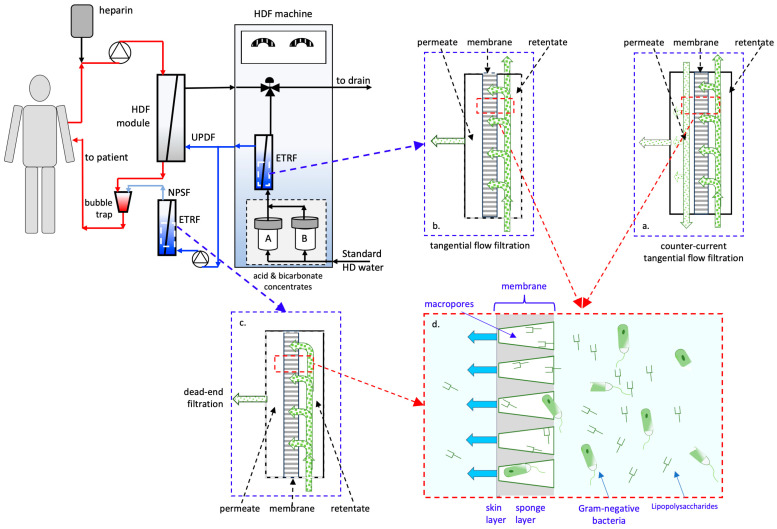
Schematic representation of: (**a**–**c**) ETRF operation; (**a**,**b**) tangential flow mode; (**c**) dead-end mode; and (**d**) the mechanisms through which ultrafiltration membranes in ETRFs remove microorganisms (e.g., Gram-negative bacteria) and toxins (e.g., lipopolysaccharides, LPS). The blue lines identify the loop where treated dialysis fluids circulate. The red lines identify the loop where blood flows. The scheme also shows where ETRFs operated in tangential flow or dead-end mode are generally positioned in the dialysate pathway of HD/HDF machines.

Table 2 shows that about half of the reusable ETRFs (6 out of 11) feature modules equipped with four headers (two on the membrane lumen side and two on the shell side). They permit counter-current tangential flow operation (Figure 2a). In such operating mode, in the retentate compartment (i.e., where the solution to be filtered flows) and in the permeate compartment (i.e., where the filtered fluid flows) fluids flow parallel to the membrane length but in opposite directions). At the same time, the permeate filters perpendicular to the membrane surface across the membrane wall either from the membrane lumen to the shell (i.e., inside–out or outwards) or from the shell to the membrane lumen (i.e., outside–in or inwards). As it flows, the retentate stream sweeps away the rejected solutes that accumulate at the membrane surface, thus minimizing concentration polarization and its detrimental effects. Three ETRFs (i.e., Baxter U8000S and U9000, and Nipro CF-609N) come with two headers on the shell side and one on the lumen side. The ETRF Diapure^®^ produced by Medica is available with one or two headers on the lumen side and two headers on the shell side. In these ETRFs, the retentate flows parallel to the membrane surface, enabling tangential flow filtration only if the solution to be filtered is fed to the compartment with two headers (Figure 2b).

The Nikkiso EF-02D and both small-scale disposable ETRFs are equipped with one header only on the membrane lumen and the shell side. In these ETRFs, filtration occurs in dead-end mode (Figure 2c). In this operating mode, both the retentate and the permeate flow perpendicular to the membrane surface.

Both small-scale ETRFs are designed to produce sterile, non-pyrogenic substitution fluid by filtering dead-end ultrapure dialysis fluid and are included in a tubing set that is easy to install on commercial HDF/HF machines.

Some of the reusable ETRFs are equipped with proprietary connectors that permit their use only, or preferentially, with/in the HDF/HF machines designed and marketed by the ETRF manufacturer. In some cases, such connectors and the ETRF-receiving cradle in the HDF/HF machine feature a design that significantly facilitates the installation of the ETRF module and its connection to the dialysis tubing.

The membrane surface area of the reusable ETRFs proposed to produce ultrapure dialysis fluid is generally similar to the modules for HDF/HF, ranging from 1 to 2.8 m^2^. Only the Nipro CF-609N is smaller, with a membrane surface area of 0.6 m^2^. The two disposable ETRFs proposed for the production of sterile non-pyrogenic substitution fluid feature a membrane surface area of 0.15 or 0.25 m^2^, about an order of magnitude lower than most reusable ETRFs.

The efficiency of bacteria or endotoxin removal/retention by an ERTF is generally expressed in terms of the logarithm base ten of the bacteria, or endotoxin, retentate-to-filtrate concentration ratio, termed the logarithmic reduction value (LRV). The LRV for the considered ETRFs ranges from LRV ≥ 7 to LRV ≥ 10 for bacteria and from LRV ≥ 2.3 to LRV ≥ 6 for endotoxins. Among the reusable ETRFs, only the Diacap^®^ Ultra by B.Braun, the Diasafe^®^ Plus by Fresenius, and the EF-02D by Nikkiso are recommended for producing online non-pyrogenic substitution fluid from ultrapure dialysate fluid in a cascade of at least two such modules.

### 3.3. Recommendations for ETRF Disinfection and Replacement

The larger ETRF modules can be cleaned, disinfected, and reused. Table 2 shows that the disinfection procedures recommended for most ETRFs use broadly varying aggressive chemicals depending on the materials used for membranes, potting, and housing. Some manufacturers recommend a rather broad spectrum of alternative disinfecting agents for their ETRFs to make the procedure fit more easily in the organization and economy of a dialysis center. The concentrations at which the use of chemicals is recommended broadly vary with the manufacturer. The use of sodium hypochlorite is recommended at concentrations from 0.05% to 4.1%; peracetic acid concentrations range from 0.02% to 0.1%; and citric acid ranges from less than 2% to 50%. Some manufacturers state the possibility of disinfecting their ETRFs with hot water. Only one manufacturer explicitly reports the possibility of coupling chemical and heat treatment. The recommended frequency of disinfection and the filter replacement schedule vary also rather broadly. The frequency of disinfection and the disinfecting agents are often tied to those recommended for the HDF/HF machine in/with which the filters are used. Concerning an average HD treatment of three hours, it may be estimated that the maximal recommended operating time of the considered ETRFs ranges approximately from 150 to 750 h, corresponding to 50–250 HD treatments. Such figures ought to be taken with caution because the actual replacement schedule depends on the quality of the dialysis water and the concentrates, as well as on the disinfecting agents and procedures and the frequency of disinfection.

## 4. Critical Analysis of ETRF Performance

### 4.1. Technical Challenges in UPDF and NPSF Production

Bacteria and endotoxin removal from pharmaceutical and biotechnological preparations have long been a key downstream bioseparation process to prevent undesired side effects in the administration of such products. More so today because many Gram-negative bacteria are used for the production of recombinant DNA products. The increasing production and use of drugs and recombinant products have made the market of endotoxin retentive filters steadily increase in the last few years. In 2023, the ETRFs market was valued at USD 350 million, and it is projected to grow to approximately USD 650 million at a Compound Annual Growth Rate (CAGR) of 7.2% [36]. One of the main drivers of such growth is the increase in chronic diseases and the market for the contaminant-free pharmaceuticals needed for their care.

Within this framework, the market of devices to remove contaminants from dialysis fluids has long lagged behind. The quality of dialysis fluids has not been paid due attention, assuming that the membranes in the hemodialyzer would hinder the passage of contaminants from dialysis fluids into the patient’s blood. Starting in the 1990s, evidence has been gathered showing that endotoxins and their fragments could even cross the wall of low-flux homogeneous cellulosic membranes [8]. It has also become clear that bacteria could grow in reverse osmosis water, due to the suboptimal design and maintenance of the water treatment and distribution systems in dialysis centers and the concentrates used for the online preparation of dialysis fluid [3,37]. The technique that has proven most effective in preventing bacteremia and endotoxemia (i.e., bacteria- and endotoxin-associated infections) caused by HD treatments is to ultrafilter the dialysis fluid upstream from the hemodialyzer just before it enters the dialysate compartment. In the following, we briefly discuss why the production of ultrapure dialysis fluid (i.e., the dialysis fluid ultrafiltrate) according to this technique is indeed a challenging task.

Briefly, endotoxins are lipopolysaccharides (LPSs) present in the cell walls of Gram-negative bacteria and contribute to cell wall organization and stability. Endotoxins consist of a core polysaccharide, a long-chain polysaccharide (termed O-antigen), and a non-polar lipid (termed Lipid A). The core polysaccharide and the O-antigen consist of repeating oligosaccharide subunits and are hydrophilic. Lipid A is a phosphorylated N-acetylglucosamine disaccharide containing a hydrophobic aliphatic fatty acid that anchors the endotoxin into the bacterial membrane. Phosphate groups and amino acids in the core oligosaccharide confer a negative charge to LPSs. Lipid A is the toxic component of LPSs because it triggers the production of pro-inflammatory cytokines and activates the coagulation cascade [38]. Exposure to high concentrations, or systematic exposure to concentrations as low as 1 ng/mL, of Lipid A elicits systemic inflammatory reactions that may cause a pyrogenic reaction and may lead to sepsis, septic shock, and even death [39]. Endotoxins are continuously shed in the environment as bacteria die, as is the case when bacteria-containing fluids are sterilized, but also during growth, division, or in case of mechanical damage or lysis. Endotoxins are amphiphilic molecules (i.e., they exhibit both hydrophobic and hydrophilic domains), bear a net negative charge in solution, are heat-resistant, and tend to aggregate (depending on pH, salt concentration, surfactants, etc.) in aqueous solution. Their hydrophobicity causes their strong affinity for hydrophobic materials. Endotoxins in their monomeric form have an average molecular weight (MW) of 10,000–30,000, with minimum MW of 2500 and maximum MW of 70,000, due to the variability in the oligosaccharide chain. Endotoxin separation from aqueous solutions is complicated by their broad molecular weight distribution, characterized by small fragments, particularly at low concentrations, and their ability to aggregate, forming micelles or vesicles with an MW greater than 1,000,000, and to interact with proteins, because of non-polar weak bonds between lipid chains and polar bonds among phosphate groups mediated by divalent cations [38,39]. The scarce efficacy of techniques based on one bioseparation mechanism only to remove endotoxins from pharmaceutical preparations has promoted the development of industrial techniques coupling more bioseparation mechanisms [39].

### 4.2. Mechanisms of Pathogen Removal

All considered ETRFs are reported to deliver better separation performance than the minimal performance recommended for bacteria and endotoxin removal, with LRV significantly higher than the minimal value required for UPDF or NPSF production. Such performance is obtained by coupling microorganism and endotoxin filtration and adsorption (Figure 2d). Operating at least two ETRFs in a cascade permits overcoming the performance limits of a single ETRF and producing sterile non-pyrogenic substitution fluid from ultrapure dialysis fluid.

Each manufacturer has developed a proprietary optimal solution for the online production of UPDF and NPSF that employs pathogen filtration and adsorption to varying extents. All ETRFs are outfitted with asymmetric ultrafiltration membranes. Most of these membranes have a single skin layer on the lumen surface (only one membrane has a second skin layer on the outer surface), measuring a fraction of a micron thick with an NMWCO lower than the maximum MW of monomeric endotoxins. The low hydraulic resistance of the thin skin layer to water enables the filtration of dialysis fluid at the high flow rates necessary for HDF treatment, with transmembrane pressure drops and membrane surface areas comparable to those of HD membrane modules. This allows the use of housings and headers designed for HD modules, thus reducing the production cost of ETRFs. The NMWCO ensures that most microorganisms and endotoxins are rejected by the membrane and cannot pass into the permeate. No information is available on the sieving coefficient spectrum (i.e., the percentage of solute that convectively crosses the membrane as a function of solute MW) of the membranes used in ETRFs. Ultrafiltration membranes generally feature a characteristic sigmoidal sieving coefficient spectrum with a slope that, in operation, becomes more slanted by the occurrence of concentration polarization phenomena. For this reason, even endotoxins with a higher MW than the membrane NMWCO may pass across the membrane wall. More so in dead-end filtration and at high endotoxin concentrations. Adsorption on the membrane pore surface decreases the endotoxin burden on the membrane selective skin layer, if the fluid to be treated flows inwards through the sponge layer first, or removes the endotoxins from the permeate stream passing through the selective skin layer, if the solution to be treated flows outwards through the skin layer first. All ETRFs are outfitted with membranes made of a hydrophobic polymer (i.e., PSu) on which endotoxins are adsorbed via interactions with the hydrophobic domains on their structure. A low membrane hydraulic resistance to aqueous solutions is obtained by blending PSu with a hydrophilic polymer (e.g., polyvinylpyrrolidone, PVP). The final outcome depends on the hydrophobicity of the polysulfone used, the pore size distribution of the membrane skin, and the required hydraulic permeability according to the Laplace equation. In the Toraysulfone^®^ membranes, the PVP molecules are chemically cross-linked to hinder their wash-out and the consequent worsening of membrane water permeability during their use. It is worth recalling that endotoxins are mainly adsorbed on the surface of the macropores in the membrane sponge layer. A large adsorption surface area in the sponge layer is generally obtained by using membranes with a wall characterized by small spherical macrovoids. Such sponge morphology confers the membranes a large adsorption capacity even if the sponge is as thick as that of HD membranes. Moreover, the large number of polymer strands bordering the spherical macrovoids increases membrane tortuosity. This further enhances the resistance to the transport of microorganisms and endotoxins across the sponge layer and their removal from the permeate stream [40]. Only in the Nipro CF-609N, the membrane adsorption capacity is enhanced by increasing the sponge layer thickness by a factor of 3–4 with respect to the membranes used in other ETRFs. Correspondingly, the membrane surface area in the CF-609N is a factor 2–4 lower than the other ETRFs, evidencing the important role played by endotoxin adsorption in UPDF preparation.

Disposable ETRFs with a smaller membrane surface area than those used to prepare UPDF are proposed to prepare sterile NPSF from UPDF with similar efficiency to the larger ETRFs. Accounting for the actual flow rate at which NPSF is produced, the lower pathogen burden and the membrane separation properties show that, in such ETRFs, the UPDF is treated with the same filtration and adsorption efficiency (e.g., the same selectivity of the skin and the same solute contact time with the polymer sponge) as in the larger ETRFs. The use of a larger reusable ETRF as the second ultrafilter in the cascade offers a redundant membrane surface area for adsorption and permits the repeated use of the ETRF. The convenience and sustainability of either solution depend on the economic framework and health assistance system of the country where the dialysis center operates and on center management.

The manufacturers’ recommendations on how to operate ETRFs vary broadly. The membranes used in an ETRF play a central role in removing contaminants from the dialysis fluids. Microorganisms and endo(myco)toxins are filtered out of the permeating dialysis fluids mainly by the membrane skin layer, whereas they are removed by adsorption mainly in the membrane sponge layer. The two processes influence one another. Fluid filtration influences the concentration of microorganisms and endotoxins at the interface between the membrane skin and the retentate. These concentrations drive pathogen adsorption at the membrane polymer surface. But in the case of membranes with two skin layers, the order in which the two processes occur influences the efficiency of contaminant removal from dialysis fluids. The extent depends on factors such as the contaminant burden, the selectivity of the skin layer, the intrinsic adsorption capacity of the membrane polymer(s), the morphology and thickness of the sponge layer, the permeate velocity, the accumulation of rejected contaminants at the skin layer, and the contact time between contaminants and the polymer surface in the sponge layer, among others. When the dialysis fluid is first filtered across the skin layer, operating the ultrafiltration membrane module in tangential filtration minimizes the accumulation of the rejected contaminants and the consequent worsening of membrane selectivity for endotoxins. Yet, for 7 out of the 13 considered ETRFs, the manufacturers recommend dead-end operation. This operating mode avoids permeate back-filtration, caused by the high membrane hydraulic permeability and the slender module design, and the associated convective transport of microorganisms and endotoxins across the membrane. It avoids the complex piping and pumping that would be needed for using ETRFs. Lastly, it decreases the contaminant burden on the sponge layer. This way, sponge layers with a high specific surface area efficiently remove from the dialysis fluid (vs. the UPDF) the residual contaminants crossing the skin layer, and the fluid easily meets the requirements for UPDF (vs. NPSF) production. In the opposite direction, the dialysis fluid flows first across the adsorbing sponge layer. This may reduce the burden of contaminants on the skin layer and may enhance its separation and filtration efficiency, but to a lesser extent in membranes with a sponge layer exhibiting large finger-like macropores. However, the absence of a sweep retentate stream could cause severe concentration polarization effects and could foul the sponge with retained microorganisms and endotoxins. This could decrease the membrane’s hydraulic permeability and the effectiveness with which the membrane skin rejects bacteria and endotoxins. It could also make membrane cleaning more demanding when the ETRFs are reused.

### 4.3. Disinfection Procedures

At least two ETRFs are required to produce UPDF and NPSF for routine HD and HDF treatments. The first ETRF is often installed in the HD/HDF machine, is integrated into the dialysate line, is used for several treatments depending on its lifetime, and is disinfected with the machine after each treatment. The selection of type, dose, and treatment time of the disinfectant is critical to prevent infections in fragile HD patients and not to induce microorganism resistance with an incorrect use [41]. The selection has to compromise with possible corrosion of the HD/HDF machine and tubing materials [42] and the patient’s sensitivity towards leaching chemicals caused by excessive dosage and long treatment times [43].

The disinfectants recommended by the manufacturers are generally effective biocidals. Sodium hypochlorite is a strong oxidizing agent, denatures membrane cell proteins, and has deleterious effects on bacterial DNA. Treatment for 2 min with 12.5 ppm hypochlorite has been reported to have bactericidal effects against *Staphylococcus aureus*,\ and with 5 ppm against *Pseudomonas aeruginosa* and its biofilms [44]. Peracetic acid oxidizes the outer cell membranes of microorganisms. It has been reported to inactivate Gram-positive and Gram-negative bacteria, fungi, and yeasts in less than a minute at concentrations lower than 0.05% in the presence of organic matter [45]. The dosage and treatment time have to be increased to 0.225% and 15 min, respectively, to inactivate viruses such as poliovirus. Inactivation of bacterial spores in suspension requires up to 1% peracetic acid and 30 min of treatment time [45].

It has been reported that reprocessing dialyzers with cellulosic and polysulfone membranes with aggressive chemicals, similar to those recommended in Table 2, might significantly change the membrane’s hydraulic permeability and selectivity [46,47,48,49,50,51]. In vitro and ex vivo tests show that despite some changes in their membrane permeability and separation properties, many commercial ETRFs consistently deliver dialysis fluids meeting the requirements set by the regulatory bodies for longer times than that indicated by the manufacturers for their replacement, provided that they are disinfected according to the manufacturer’s recommendations [35,52]. Together with the requirement by ISO that conformity of dialysis fluids with the ISO23500-5:2023 “… *shall be ensured by proper operation of a validated system, verified in accordance with the manufacturer’s instructions at the time of installation, and confirmed by the user with a regular surveillance and maintenance schedule*” [27], the recommended procedures strengthen the importance that dialysis fluids, in particular, sterile non-pyrogenic substitution fluid, be produced according to a validated process and the system be used according to the manufacturer’s instructions [34].

## 5. New Challenges and Future Outlook

### 5.1. New Challenges

The commercial availability of ETRFs that effectively remove microorganisms and endotoxins from dialysis fluids has made it possible to produce online ultrapure dialysis fluids and sterile non-pyrogenic substitution fluid sustainably. This has significantly reduced the risk of infections and has enhanced the biocompatibility of renal treatments and the quality of life of millions of ESKD patients. Nonetheless, ESKD patients on HD treatment still are chronically inflamed, get infected, and some die [53,54,55,56]. The prevention of infections remains one of the simplest and most cost-effective strategies to control the occurrence of new infection outbreaks, to reduce the number of hospitalizations, to control costs, and to enhance the quality of life of ESKD patients. Effective preventive measures should include the supply of dialysis water of high microbiological and chemical quality to all dialysis centers and the availability of optimal systems to consistently produce and distribute such water to all HD/HDF machines. A brief, critical analysis of the gathered information may help figure out whether there is room for improvements in ETRF design and operation to further enhance the treatment of ESKD patients in the short-to-mid-term.

The identification of prevalent pathogens is key to optimizing HD and HDF treatments and to preventing infections during treatment. ETRF design, operation, disinfection procedures, and replacement schedules in dialysis centers are generally optimized to effectively handle microbiological challenges such as those posed by coliform Gram-negative bacteria (e.g., *E. Coli*, *Enterobacter*), Gram-positive cocci (e.g., *Staphylococcus aureus, Coagulase negative Staphylococci*), and Gram-negative bacilli [57,58]. It is generally assumed that the water treatment plant and distribution systems deliver dialysis water of good quality to the HD/HDF machines.

The analysis of the infection outbreaks of the last ten years shows that there has been a surge in deadly infections in dialysis centers caused by new waterborne opportunistic pathogens, such as non-fermentative Gram-negative *bacilli* (e.g., of the *Pseudomonas* family) [54], filamentous Gram-negative mycobacteria (e.g., the thermophilic non-tuberculous *Mycobacteria*, *Mycobacterium tuberculosis, Mycobacterium avium-intracellulare, Mycobacterium saskatchewanense)* [56,59,60,61], and *fungi* (e.g., from filamentous *fungi,* like those of the *Penicillium genus* and the *Fusarium genus*, to yeasts, like *Candida* spp.) [62,63], among others. The effects of some of these pathogens on immunocompromised and vulnerable ESKD patients have been or are under-recognized, often because of the limited diagnostic capacity in many low- and middle-income countries, the underestimation of the risk that patients run when exposed to them (e.g., ruling them out as mere contaminants), or simply because they are “*unusual*” and neglected when the quality of dialysis fluids is periodically ascertained. This is the case of non-tuberculous *Mycobacteria* [59,64], *fungi* and yeasts, bacteria of the *Ralstonia* or *Burkholderia genus* [54], or non-fermenting Gram-negative *bacilli* (e.g., *Pseudomonas aeruginosa*) [62], the concentration of which is generally not limited by international standards in the water for hemodialysis and dialysis fluids, but for a few countries (e.g., Austria, Germany, Italy, Sweden) in the case of *fungi* and yeasts [53,65].

Additional information about what has contributed to the occurrence of infection outbreaks may be guessed from the properties of these pathogens, often gathered from the few available published reports on their characterization and identification. Some of them grow rapidly; some may pass through the 0.45 and 0.2 μm filters that are used to sterilize dialysis water (e.g., the Gram-negative non-fermentative *Ralstonia* bacteria [66]) and even through the ETRF membranes used for the production of ultrapure dialysis fluid (e.g., *Mycobacterium saskatchewanense* [59]); some form biofilms (e.g., non-tuberculous *Mycobacteria, fungi, Ralstonia* bacteria, *Methylobacterium* spp., most non-fermenting Gram-negative *bacilli*); some form spores; and some have developed resistance against common antimicrobials, antifungals, and antibiotics. The first challenge to ensure a safe treatment is to detect the causing pathogen(s) and to evaluate its possible effects on patients. The fact that many of these pathogens form biofilms, in which they are embedded, complicates the estimation of the actual microbiological burden on the membrane filter used to remove them [7,8]. The elongated shape, the size, and the compliance of their wall structure may help the spores of some bacteria cross the wall of the microfiltration membranes featuring a maximal pore size of about 0.2 mm, which are often used to sterilize water or the wall of some ultrafiltration membranes, and they may contaminate the permeating dialysis fluid. Even when the membranes do not let microorganisms pass, their accumulation in the upstream compartment of ETRFs may favor the leakage of endotoxin fragments and toxic bacterial or fungal metabolites with an MW lower than the membrane NMWCO into the dialysis fluids [67,68]. Similar concerns also exist for viral disinfection. Once the viral capsid has been destroyed, nucleic acids may be released into the fluid, and some of them are infective [44]. The risk of contamination is higher when the membranes feature a slanted sieving coefficient spectrum.

Evidence suggests that infection occurrence may depend on the machine used for patient treatment [55,68] and that some infections are more likely to occur when patients are treated with machines that are more intensely used [69]. It may be expected that operation and maintenance are more critical for HD/HDF machines in which the primary ETRF is included in the dialysate pathway of the machine and is disinfected with them. In fact, as a machine, and the ETRF in it, is more frequently used, a biofilm is more likely to form at the surface of machine parts and tubing in the dialysate pathway, and even at the filter membrane, which protects the pathogens from antimicrobials and antifungals, helping them survive normal disinfection procedures. Moreover, once formed, biofilms are very difficult to eradicate. In one study, the presence of the same bacterial strain was detected in a machine for several years [55]. It would be impossible to eradicate biofilms that might form in the sponge of asymmetric membranes in which contaminated fluids are filtered dead-end first through the membrane sponge layer and then through the membrane skin layer. Effective disinfection procedures ought to account for such occurrences and take specific measures (e.g., replace the ETRF more often) to be safe and fault-proof.

Biofilm growth, spore formation, and the acquired resistance against common antimicrobials, antifungals, and antibiotics make the “*unusual*” pathogens dangerous for patients and less susceptible to disinfection procedures based on the conservative use of common biocides. Conservative means that the selected biocide dose and exposure time balance off the maximization of pathogen removal efficiency and the minimization of machine corrosion, tubing chemical degradation, and patient hypersensitivity reactions. Many “*unusual*” opportunistic pathogens, such as non-tuberculosis *Mycobacteria* [59], non-fermenting Gram-negative *bacilli* [70], and Gram-negative bacteria [44] (e.g., *P. aeruginosa*, *Ralstonia* spp., and *Burkholderia* spp.), are or have become resistant to antiseptics, disinfectants, and antibiotics. A similar emergency is also taking place due to the increasing resistance of pathogenic *fungi* (e.g., the filamentous fungi *Scedosporium* spp. and *Fusarium* spp.) against antifungals [71].

The occurrence of deadly infection outbreaks among ESKD patients on hemodialysis caused by new emerging opportunistic pathogens might be prevented with the integrated optimization of all the processes contributing to contaminant elimination. An ideal approach could include the pre-treatment assessment of the presence of possible pathogens in the HD module and dialysis fluids; their identification and characterization; the preparation of dialysis and substitution fluids as if they were pharmaceutical products [72]; the use of disposable ETRFs; and the use of disposable tubing and parts on the dialysate pathway in HD/HDF machines, to say but a few. In the short-to-mid-term, regulatory issues, the cost of labor and medical devices, and the consequent burden on healthcare systems make this impossible and not sustainable even in more developed countries.

However, efforts must be made to protect ESKD patients from the short- and long-term effects (e.g., infection and chronic inflammation) of faulty or suboptimal water treatments and distribution systems or HD/HDF disinfection procedures that let new opportunistic pathogens spread and grow.

### 5.2. Future Outlook

A few minimal adjustments to the design and operation of ETRFs and the HD/HDF machines that use them could help. In these brief considerations, the attention is only focused on the processes to which dialysis water is (or may be) subjected once it enters the HD/HDF machine.

The first consideration deals with the periodic assessment of microbial challenges and the actual bioburden in the fluids to be treated. Such an important prevention step is often skipped on account of the difficulty in evaluating the quality of dialysis fluids as they are prepared online. An effective approach could be to identify and characterize possible pathogens and their concentration in samples harvested periodically from the retentate fluids upstream from the filtering membrane [55]. This would permit us to evaluate if pathogens are present and grow in the pipeline of UPDF and NPSF production during routine operation or maintenance work and to organize a response tailored to the specific challenges posed by the identified pathogen. In the case of pathogens known for their production of low MW metabolic toxins (e.g., endotoxins or mycotoxins) and aptitude to form biofilms, a possible response could be to change the tubing, the contaminated parts of the HD/HDF machine, and/or the ETRF before that planned by the replacement schedule [55].

The design of some ETRFs equipped with asymmetric membranes could also be slightly modified. Filtering the retentate dialysis fluid (vs. ultrapure dialysis fluid) in tangential filtration mode by making it flow parallel to, and in contact with, the membrane skin layer would minimize the concentration polarization of rejected microorganisms and the toxins that they produce and membrane fouling. Provided that this does not increase the permeate flux for the occurrence of back-filtration phenomena, such an operating mode would minimize the detrimental effects associated with concentration polarization and fouling, such as biofilm formation at the membrane surface and the leakage of endotoxins and other low MW microbial/fungal metabolic toxins into the patient’s bloodstream. This approach could be particularly useful for primary ETRFs operated dead-end. Correspondingly, where needed, the HD/HDF machine should be equipped with the necessary pumping and control devices and a modified fluid pathway to permit the recirculation of dialysis fluid (for the primary ETRF) and ultrapure dialysis fluid (for the secondary ETRF). Concerning the membranes, those used in commercial ETRFs are generally made of hydrophobic PSu. PSu features a good endotoxin adsorption capacity, which permits the maximization of pathogen removal. The only separation property known for a few membranes is the NMWCO, which is reported to be in the 6000 to 30,000 range. Generally speaking, using membranes exhibiting an NMWCO in the range of a few thousand and a steep sieving coefficient spectrum curve could contribute to removing low MW microbial toxins more effectively and to a great extent, even in the presence of strongly contaminated water.

As set by current norms, the quality of ultrapure dialysis and sterile non-pyrogenic substitution fluid is guaranteed when ETRFs and HD/HDF machines are operated and disinfected as recommended by the manufacturer. Generally, such norms shift the burden of control of the quality of dialysis fluids onto the operators of dialysis centers. This is the case as long as the procedures recommended by the manufacturers are used against the reference microbiological pathogens/contaminants for which they are optimized. As new opportunistic pathogens, often resistant to common biocides, contaminate the dialysis fluids, the recommended procedures may end up being only partially effective or even ineffective. In such cases, in the interest of patients, dialysis centers and manufacturers should join forces and work together to adapt the recommended procedures or to develop new, more effective disinfection procedures to remove or kill the new contaminants. The most immediate response that could be envisioned is to exploit more than one mechanism to kill the identified pathogens. In suspect cases, as the first line of defense, the disinfection procedure could combine a chemical treatment with an adequate dose of a selected biocide and a treatment based on the administration of energy to kill the pathogens (e.g., a thermal treatment) [45]. Indeed, chlorination in combination with thermal treatment at 60 °C is recognized as an effective disinfection measure. Treatments with 99% acetic acid or with peracetic acid performed at 80 °C have been reported to effectively disinfect HD distribution pipes and remove the biofilms formed at their surface [73]. More recently, treatment with a citrothermic procedure has been reported to effectively disinfect dialysis machines contaminated by *M. saskatchewanense* mycobacteria, for which treatment with peracetic acid only had been unsuccessful [60]. Although most manufacturers claim that their ETRFs may be heat-treated, today, the combination of chemical and thermal treatment is prospected by only one out of the seven contacted manufacturers to disinfect their ETRFs. In the case of infections of microorganisms forming difficult-to-eradicate biofilms, which are life-threatening because of the toxicity of the low MW metabolic toxins that they release, the last line of defense could be to replace all infected parts of the HD/HDF machines and the dialysate pathway [56], and even the contaminated pipes and parts of the water distribution and treatment systems [55].

No report was found to associate the occurrence of infection outbreaks with poor performance, or failure, of secondary ETRFs in the production of sterile non-pyrogenic substitution fluid. The fact that such ETRFs are frequently replaced, and about half of those commercially available are disposable and supplied already included in the recirculation loop for the ultrapure dialysis fluid, possibly contributes to such an outcome. An interesting and relatively easy-to-implement preventive measure, in addition to those above, could also be to supply the primary ETRFs already included in a dialysate/recirculation path/loop set and use them in a disposable fashion. This would require changes to the HD/HDF machines in which the ETRFs are used and also to the ETRFs, with additional costs. However, the cost reduction in hospitalization, control visits, and drugs, and the economic gain of society at large for the improved quality of life and the quicker return to a productive life of ESKD patients, might outweigh such additional costs in the mid-to-long term.

In the long term, it would be worthwhile to also consider the possibility of developing membranes more resistant to chemicals and high-energy treatments (e.g., nanofiltration ceramic membranes [74]) that would permit the development of optimal disinfection treatments tailored to the properties of new opportunistic pathogens that account for all the factors that may protect the pathogens (e.g., the regrowth promoting effect of nutrients [75]).

## 6. Conclusions

The current norms for the production of ultrapure dialysis and substitution fluids state that fluid quality is ensured if ETRFs and HD/HDF machines are disinfected and maintained according to the validated procedures recommended by the manufacturers. These norms make it of paramount importance for the operators of dialysis centers to carefully select the commercial ETRFs that most conveniently fit in the disinfection and maintenance procedures and the logistic and economic organization of a dialysis center. Unfortunately, gathering information about the specifications and characteristics of commercial ETRFs and their maintenance requirements is not easy.

To bridge this gap, in this paper, the most relevant properties of some commercial ETRFs are reported in a table format that may be quickly and easily consulted. To enable a rational ETRF selection, the properties of the selected ETRFs are also critically reviewed. The analysis of recent infection outbreaks suggests that new opportunistic pathogens are emerging against which current membrane filters and disinfection procedures are less effective than expected. Little improvements to ETRF and HD/HDF machine design and operation and the targeted use of disinfection procedures might help relieve the dangerous effects of infections on ESKD patients caused by new opportunistic pathogens in the short-term and in a cost-effective fashion.

## Figures and Tables

**Table 1:** Requirements of fluids for hemodialysis (HD), hemofiltration (HF), and hemodiafiltration (HDF) set by international regulatory bodies. Legend: *AAMI*—Association for the Advancement of Medical Instrumentation; *ANSI*—American National Standards Institute; *dialysis fluid*—dialysate prepared by diluting the acid/base concentrate with dialysis water and delivered to the HD/HDF/HF membrane modules; *dialysis water*—treated water entering the HD/HDF/HF machine; *endotoxin concentration*—endotoxin unit, EU; *microbial count*—colony-forming unit; CFU; *ISO*—International Organization for Standardization; *JSDT*—Japanese Society for Dialysis Treatment; *NPSF*—sterile non-pyrogenic substitution fluid; *UPDF*—ultrapure dialysis fluid. * Tested according to paragraph 5, Section 5, of ISO 23500-5:2024.

	ISO/ANSI/AAMI		JSDT	
	Microbial Count (CFU/mL)	Endotoxin Concentration (EU/mL)	Microbial Count (CFU/mL)	Endotoxin Concentration (EU/mL)
Dialysis water	<100 *	<0.25 *	<100	<0.05
Dialysis fluid	<100 *	<0.5 *	<100	<0.05
UPDF	<0.1 *	<0.003 *	<0.1	<0.001
NPSF	Sterile	Non-pyrogenic	Sterile	Non-pyrogenic

**Table 2:** Commercial reusable membrane endotoxin retentive filters (ETRFs). Legend: HD—hemodialysis; LRV = logarithmic reduction value (Log 10 (concentration in challenge/concentration in filtrate); N.A.—not available; NMWCO—nominal molecular weight cut-off; PA—polyamide; PVP—polyvinylpyrrolidone; TMP—transmembrane pressure (i.e., the pressure difference between retentate and permeate compartment). ^1^ From the 09/16 brochure. ^2^ Challenge: *E. coli* O55:B5 endotoxin (Whittaker, USA). ^3^ Challenge: *Pseudomonas diminuta* ATCC 19146. ^4^ Challenge: 0.9% sodium chloride at 37 °C.

Reusable Endotoxin Retentive Filters (ETRFs)			
**Manufacturer**	Baxter International Inc. USA	Baxter International Inc. USA	Baxter International Inc. USA
**Module tradename**	Diaclear^®^ Ultrafilter	U8000 S Ultrafilter	U9000 Ultrafilter
**Module image**	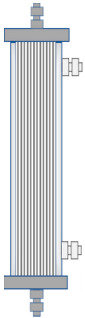	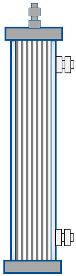	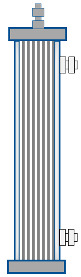
**Url for information**	https://renalcare.baxter.com/sites/g/files/ebysai1471/files/2019-12/Diaclear_datasheet_2019_A4_GLBL_MG204_19-0002_low.pdf (accessed on 30 January 2025)	https://renalcare.baxter.com/sites/g/files/ebysai1471/files/2019-12/U8000S_datasheet_2019_A4_EUMP_MG204_16-0002%282%29_low.pdf (accessed on 30 January 2025)	https://renalcare.baxter.com/sites/g/files/ebysai1471/files/2019-12/U9000_datasheet_2019_A4_EUMP_MG204_16-0001%281%29_low.pdf (accessed on 30 January 2025)
**Module design and operation**			
**Indications for use**	Purification of dialysis fluid	Purification of incoming water for dialysis fluid and purification of dialysis fluid	Purification of incoming water for dialysis fluid and purification of dialysis fluid
**Membrane arrangement**	Shell-and-tube	Shell-and-tube	Shell-and-tube
**Operating mode**	Cross-flow/Dead-end	Cross-flow/Dead-end	Cross-flow/Dead-end
**Permeate direction** **(outwards vs. inwards)**	N.A.	N.A.	N.A.
**Recommended HD machine(s)**	Only Baxter HD machines equipped with Diaclear holder	Only Baxter HD machines equipped with U8000 holder	Only Baxter HD machines equipped with ultrafilter holder
**Module connectors** **(number/type)**	4×/Only Diaclear holders	3×/Only U8000 holders	3×/Only ultrafilter holders
**Membrane specifications**			
**Geometry**	Hollow Fiber	Hollow Fiber	Hollow Fiber
**Material chemistry**	Polyarylethersulfone (PAES)	Polyarylethersulfone (PAES/PVP/PA)	Polyarylethersulfone (PAES/PVP)
**Wall structure**	N.A.	N.A.	N.A.
**Selective skin layer(s)**	N.A.	N.A.	N.A.
**Wall pore structure**	N.A.	N.A.	N.A.
**NMWCO, Da**	N.A.	N.A.	N.A.
**Wall thickness, mm**	50 ^1^	N.A.	N.A.
**Inner diameter, mm**	215 ^1^	N.A.	N.A.
**Filtration coefficient, ** **mL/(h mmHg)**	500 ^1,4^	N.A.	N.A.
**Module specifications**			
**Sterilization technique**	g rays	Steam	N.A.
**Filtration area, m^2^**	1.3	2.1	2.4
**Number of fibers**	8300 ^1^	N.A.	N.A.
**TMP_max_**	200 mmHg ^1^	N.A.	N.A.
**Endotoxin retention**	LRV > 3.5 ^2^	LRV > 2.3 ^2^	LRV > 3.5 ^2^
**Bacteria retention**	LRV > 7 ^3^	LRV > 7 ^3^	LRV > 7 ^3^
**Recommendations for replacement and disinfection**			
**Recommended filter replacement schedule**	Replace after the following: - A total of 50 HD sessions. - A total of 100 heat disinfections or depending on the results of microbiological controls of ultrapure dialysis fluid.	Replace routinely as follows: - Every month. - After max 2 months of use—depending on results of microbiological controls of ultrapure dialysis fluid.	Replace after the following: - Three months of use. - A total of 150 disinfection cycles. - Max 12 disinfections with sodium hypochlorite over filter lifetime.
**Disinfection schedule**	Disinfect w/HD machine	Disinfect w/HD machine	Disinfect w/HD machine
**Compatible disinfection agents**	Peracetic acid (≤0.1%) Acetic acid (≤1%) Sodium hypochlorite (≤0.5%) Citric acid (≤2%) Compatible w/heat disinfection	Peracetic acid (≤0.1%) Sodium carbonate (≤2.0%) Sodium hypochlorite (≤0.5%) Citric acid (≤2.0%) Compatible w/heat disinfection	Peracetic acid (≤0.1%) Sodium carbonate (≤0.5%) Sodium hypochlorite (≤0.5%) Citric acid (≤2.0%) Compatible w/heat disinfection
**Frequency of disinfection**	According to program of HD machine	According to program of HD machine	According to program of HD machine

**Table 3:** Commercial reusable membrane endotoxin retentive filters (ETRFs). Legend: HD—hemodialysis; HDF—hemodiafiltration; HF—hemofiltration; LRV = logarithmic reduction value (Log 10 (concentration in challenge/concentration in filtrate); N.A.—not available; NMWCO—nominal molecular weight cut-off; TMP—transmembrane pressure (i.e., the pressure difference between retentate and permeate compartment); tmts—treatments.

Reusable Endotoxin Retentive Filters (ETRFs)			
**Manufacturer**	B.Braun Avitum AG Germany	Dialife SA Switzerland	Fresenius Medical Care (FMC) AG Germany
**Module tradename**	Diacap^®^ Ultra	Ultradia 210	Diasafe^®^ Plus
**Module image** (images shown with permission)	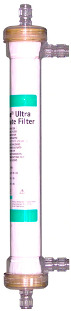	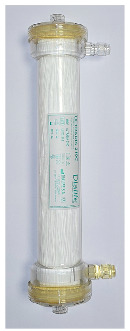	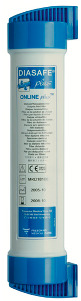
**Url for information**	http://www.knck.co.kr/down/technical_data_diacap_ultr.pdf (accessed on 30 January 2025)	https://www.dialifegroup.com/products/endotoxin-filters/ (accessed on 30 January 2025)	https://www.freseniusmedicalcare.it/it/professionisti-sanitari/emodialisi/dializzatori/filtri-del-liquido-di-dialisi-diasafeplus (accessed on 30 January 2025)
**Module design and operation**			
**Indications for use**	Preparation of high-purity dialysis fluid (1×) and sterile and depyrogenated substitution fluid (2×) for online HDF/HF	Depyrogenation filter for the preparation of high-purity dialysate	Production of ultrapure dialysis fluid (1×) and of substitution fluid for online HDF/HF therapies
**Membrane arrangement**	Shell-and-tube	Shell-and-tube	Shell-and-tube
**Operating mode**	Cross-flow/Dead-end	Cross-flow/Dead-end	Cross-flow/Dead-end
**Permeate direction** **(outwards vs. inwards)**	N.A.	Outwards	Inside–out (outwards)
**Recommended HD machine(s)**	Only B.Braun Dialog^+®^ or Dialog iQ^®^ HD machines	DIANOVA X1, DIANOVA X2	FMC compatible HD machines
**Module connectors** **(number/type)**	4×/Hansen connectors	4× Hansen/twist lock connectors	4×/Diafix^TM^ quick lock system
**Membrane specifications**			
**Geometry**	Hollow Fiber	Hollow Fiber	Hollow Fiber
**Material chemistry**	Polysulfone (PSu)	Medisulfone^®^ UF (PSu)	Fresenius polysulfone^®^ (PSu)
**Wall structure**	N.A.	Asymmetric	Asymmetric
**Selective skin layer(s)**	N.A.	Inner skin	N.A.
**Wall pore structure**	N.A.	finger-like macropores	spherical macrovoids
**NMWCO, Da**	N.A.	15,000	N.A.
**Wall thickness, mm**	40	50	35
**Inner diameter, mm**	200	250	185
**Filtration coefficient, ** **mL/(h mmHg)**	270	302	300
**Module specifications**			
**Sterilization technique**	g rays	Ethylene Oxide (EtO)	Steam
**Filtration area, m^2^**	1.2	2.1	2.2
**Number of fibers**	N.A.	7500	17,000
**TMP_max_**	500 mmHg	600 mmHg	1500 mmHg (2 bar)
**Endotoxin retention**	≥10^6^ IU/mL	≥10^5^	LRV > 6
**Bacteria retention**	≥10^6^ IU/mL	≥10^10^	N.A.
**Recommendations for replacement and disinfection**			
**Recommended filter replacement schedule**	Replace after the following: - A total of150 treatments. - A total of 900 h of operation. - Upon failing the automatic integrity test.	Replace after 900 h (machine working hours)	Max 1–3 treatments/day. Replace after the following: - A total of 12 weeks/ - A total of 100 treatments. - A total of 11 hypochlorite tmts.
**Disinfection schedule**	N.A.	On a daily basis	N.A.
**Compatible disinfection agents**	50% citric acid @ 83 °C (citrothermal treatment) w/Dialog iQ^®^ HD machines Tiutol (hypochlorite 4.1% @ 60 °C) (compatible, not recommended) only at ETRF exchange and w/Dialog^+®^ HD machines	Citric Acid 50% (recommended) Peracetic acid Hydrogen peroxide (compatible, not recommended)	Puristeril^®^ 340/plus Puristeril^®^ plus Diasteril^®^ or Citrosteril^®^ Water @84 °C Sporotal^®^100 (max 11 tmts)
**Frequency of disinfection**	Before each treatment w/50% citric acid @ 83 °C and at any cleaning or disinfection cycle performed on the HD machine	Once a day (HD) After each treatment (HDF)	**Daily use machines:** -After each treatment (ordinary disinfection). -After each week (hypochlorite, ordinary sterilization). **Non-daily use machines:** - Every 3 days (ordinary disinfection)/ - Every week (hypochlorite, ordinary sterilization).

**Table 4:** Commercial reusable membrane endotoxin retentive filters (ETRFs). Legend: HD—hemodialysis; HDF—hemodiafiltration; HF—hemofiltration; LRV = logarithmic reduction value (Log 10 (concentration in challenge/concentration in filtrate); N.A.—not available; NMWCO—nominal molecular weight cut-off; PES—polyethersulfone; PVP—polyvinylpyrrolidone; TMP—transmembrane pressure (i.e., the pressure difference between retentate and permeate compartment). ^1^ From [35]; ^2^ by similarity to the HD membranes of the same firm.

Reusable Endotoxin Retentive Filters (ETRFs)			
**Manufacturer**	Medica Group Italy	Nikkiso Europe Japan	Nikkiso Europe Japan
**Module tradename**	DiaPure^®^	EF-01D (wet)	EF-02D (dry)
**Module image** (images shown with permission)	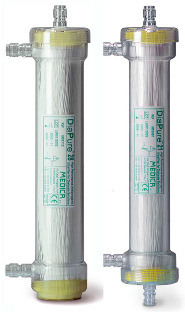	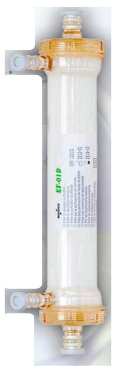	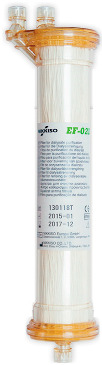
**Url for information**	https://www.medica-spa.com/business-units/prod/purificazione-acqua/filtri-medicali/ultrafiltri-diapure (accessed on 30 January 2025)	https://www.nikkiso.com/products/medical/products/dialyzers_filters.html (accessed on 30 January 2025)	https://www.nikkiso-europe.eu/en/products/dialyzers-filters-for-dialysate-purification/filters-for-dialysate-purification (accessed on 30 January 2025)
**Module design and operation**			
**Indications for use**	Anti-pyrogenic ultrafilter for dialysate fluids	Preparation of ultraclean dialysis fluid and substitution fluid for HDF/HF in combination with EFL-015 system	Preparation of ultraclean dialysis fluid (1×) and substitution fluid for HDF/HF (2× in series or 1× with EFL-015 system)
**Membrane arrangement**	Shell-and-tube	Shell-and-tube	Shell-and-tube
**Operating mode**	Dead-end/Cross-flow	Cross-flow/Dead-end	Dead-end
**Permeate direction** **(outwards vs. inwards)**	Outside–in (inwards)	Outside–in and Inside–out (inwards and outwards)	Outside–in (inwards)
**Recommended HD machine(s)**	N.A.	Nikkiso DBB-03/-05 (until SN 603132-04)	Nikkiso DBB-05 (from SN 603133-01)/-06/-07 Nikkiso DBB-EXA/ES
**Module connectors** **(number/type)**	3× or 4×/N.A.	4×/Connection ports comply with “Connection for dialysis fluid” in JIS T3250:2005	2×/Nikkiso EF-02 holders for easy filter replacement
**Membrane specifications**			
**Geometry**	Hollow Fiber	Hollow Fiber	Hollow Fiber
**Material chemistry**	Medisulfone^®^ (PSu)	Hydrophobic PEPA^®^ (Polyester-polymer alloy) (PES and polyarylate)	Hydrophobic PEPA^®^ (Polyester-polymer alloy) (PES and polyarylate)
**Wall structure**	Asymmetric	Asymmetric, three-layer structure ^2^	Asymmetric, three-layer structure ^2^
**Selective skin layer(s)**	Inner skin	Outer and inner skin ^2^	Outer and inner skin ^2^
**Wall pore structure**	finger-like macropores	spherical macrovoids ^2^	spherical macrovoids ^2^
**NMWCO, Da**	15,000	N.A.	30,000 ^1^
**Wall thickness, mm**	N.A.	30	50
**Inner diameter, mm**	N.A.	210	210
**Filtration coefficient, ** **mL/(h mmHg)**	400/300	580	480
**Module specifications**			
**Sterilization technique**	Ethylene oxide (ETO)	g rays	g rays
**Filtration area, m^2^**	2.8/2.1	1.2	1.0
**Number of fibers**	N.A.	N.A.	N.A.
**TMP_max_**	N.A.	735 mmHg (98 kPa)	735 mmHg (98 kPa)
**Endotoxin retention**	LRV ≥ 5	LRV ≥ 3	LRV ≥ 3
**Bacteria retention**	LRV ≥ 10	LRV ≥ 8	LRV ≥ 8
**Recommendations for replacement and disinfection**			
**Recommended filter replacement schedule**	Replace after the following: - Max 1 to 2 months of use depending on water quality.	Replace after 750 h of operation	Replace after 750 h of operation
**Disinfection schedule**	N.A.	Refer to HD machine	Refer to HD machine
**Compatible disinfection agents**	N.A.	Citric acid Peracetic acid Sodium hypochlorite	Citric acid Peracetic acid Sodium hypochlorite
**Frequency of disinfection**	N.A.	Refer to HD machine	Refer to HD machine

**Table 5:** Commercial reusable membrane endotoxin retentive filters (ETRFs). Legend: HD—hemodialysis; HDF—hemodiafiltration; HF—hemofiltration; LRV = logarithmic reduction value (Log 10 (concentration in challenge/concentration in filtrate); N.A.—not available; NMWCO—nominal molecular weight cut-off; PES—polyethersulfone; TMP—transmembrane pressure (i.e., the pressure difference between retentate and permeate compartment). ^1^ By similarity to the HD membranes of the same firm.

Reusable Endotoxin Retentive Filters (ETRFs)		
**Manufacturer**	Nipro Corporation Japan	Toray Industries, Inc. Japan
**Module tradename**	CF-609N	TE-12R
**Module image** (image shown with permission)	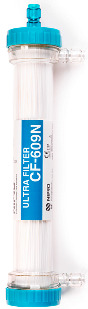	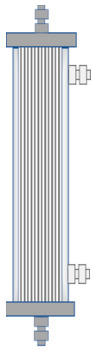
**Url for information**	https://www.nipro.co.jp/en/assets/document/business/cf-609n.pdf (accessed on 30 January 2025)	https://www.toray-medical.com/en/products/dialysis/dia_0110.html (accessed on 30 January 2025)
**Module design and operation**		
**Indications for use**	Endotoxin retention filter	Endotoxin-retentive filter
**Membrane arrangement**	Shell-and-tube	Shell-and-tube
**Operating mode**	Cross-flow/Dead-end	Cross-flow/Dead-end
**Permeate direction** **(outwards vs. inwards)**	Outside–in ^1^ (inwards)	Inside–out (outwards)
**Recommended HD machine(s)**	N.A.	Toray HD machines
**Module connectors** **(number/type)**	3×/N.A.	4×/N.A.
**Membrane specifications**		
**Geometry**	Hollow fiber	Hollow fiber
**Material chemistry**	Hydrophobic polyethersulfone (PES)	Polysulfone (PSu)
**Wall structure**	N.A.	Asymmetric
**Selective skin layer(s)**	N.A.	Inner skin
**Wall pore structure**	N.A.	Spherical macrovoids
**NMWCO, Da**	6000	N.A.
**Wall thickness, mm**	150	60
**Inner diameter, mm**	500	200
**Filtration coefficient, ** **mL/(h mmHg)**	590	N.A.
**Module specifications**		
**Sterilization technique**	g rays	g rays
**Filtration area, m^2^**	0.6	1.2
**Number of fibers**	N.A.	N.A.
**TMP_max_**	1103 mmHg (147 kPa)	495 mmHg (66 kPa)
**Endotoxin retention**	LRV ^1^ ≥ 3	LRV ≥ 3
**Bacteria retention**	N.A.	LRV ≥ 8
**Recommendations for replacement and disinfection**		
**Recommended filter replacement schedule**	N.A.	Replace after 80–100 treatments or 3 months
**Disinfection protocol**	N.A.	N.A.
**Compatible disinfection agents**	N.A.	Hot water (<85 °C) Sodium hypochlorite (0.05–0.1%) acetic acid (<1%) peracetic acid (0.02–0.04%) all for 20–60 min Citric acid (≤2%) can be coupled with hot water

**Table 6:** Commercial disposable membrane endotoxin retentive filters (ETRFs). Legend: HD—hemodialysis; HDF—hemodiafiltration; HF—hemofiltration; LRV = logarithmic reduction value (Log 10 (concentration in challenge/concentration in filtrate); N.A.—not available; NMWCO—nominal molecular weight cut-off; TMP—transmembrane pressure. (i.e., the pressure difference between retentate and permeate compartment). ^1^ By similarity to HD membranes of the same firm.

	Disposable Endotoxin Retentive Filters (ETRFs)	
**Manufacturer**	Medica Group Italy	Nikkiso Europe Japan
**Module tradename**	D150/W	EFL-015
**Module image** (images shown with permission)	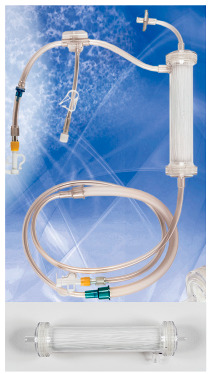	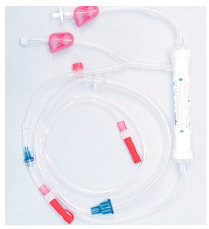
**Url for information**	https://www.medica-spa.com/business-units/prod/medicale/dialisi/hdf-on-line (accessed on 30 January 2025)	https://www.nikkiso-europe.eu/fileadmin/downloads/blutschlauchsysteme/BTL_DBB-XX/NIKKISO_AV-06_and_Online_sets_brochure.pdf (accessed on 30 January 2025)
	**Module design and operation**	
**Indications for use**	Removal of pyrogens and endotoxins from water solutions such as replacement solutions for online HDF	Online preparation of substitution fluid for HDF/HF in series to Nikkiso EF-02D reusable ETRF
**Membrane arrangement**	Shell-and-tube	Shell-and-tube
**Operating mode**	Dead-end	Cross-flow/Dead-end
**Permeate direction** **(outwards vs. inwards)**	Outside–in (inwards)	Inside–out (Outwards)
**Recommended HD machine(s)**	Provided with tubing set to produce online substitution fluid for HDF/HF	Nikkiso HD machines DBB-03/-05/-07
**Module connectors** **(number/type)**	Inlet and vent port are female luer lock, solution outlet port is male luer lock	Universal connectors
	**Membrane specifications**	
**Geometry**	Hollow fiber	Hollow fiber
**Material chemistry**	Medisulfone^®^ (PSu)	PEPA^®^ (Polyester-polymer alloy) (PES and polyarylate)
**Wall structure**	Asymmetric	Asymmetric, three-layer structure ^1^
**Selective skin layer(s)**	Inner skin	Inner and outer skin ^1^
**Wall pore structure**	Finger-like macrovoids	Spherical macrovoids ^1^
**NMWCO, Da**	15,000	N.A.
**Wall thickness, mm**	50	N.A.
**Inner diameter, mm**	250	N.A.
**Filtration coefficient, ** **mL/(h mmHg)**	90	N.A.
	**Module specifications**	
**Sterilization technique**	Supplied non-sterile It may be sterilized with Ethylene oxide (ETO), b-/g-rays	ETO
**Filtration area, m^2^**	0.25	0.15
**Number of fibers**	N.A.	N.A.
**TMP_max_**	500 mmHg	N.A.
**Endotoxin retention**	LRV ≥ 5	N.A.
**Bacteria retention**	LRV ≥ 10	N.A.
	**Recommendations for replacement and disinfection**	
**Recommended filter replacement schedule**	Disposable	Disposable
**Disinfection protocol**	Not applicable	Not applicable
**Compatible disinfection agents**	Not applicable	Not applicable

## Data Availability

No new data were created or analyzed in this study. Data sharing is not applicable to this article.
